# Supplementary material for: Role of Different Members of the AGPAT Gene Family in Milk Fat Synthesis in Bubalus bubalis
Source: Genes (Basel). 2023 Nov 13;14(11):2072. doi: 10.3390/genes14112072 (PMC10671497; doi:10.3390/genes14112072)
Supplement: Supplementary file 1 [file genes-14-02072-s001.zip › genes-2679713-supplementary.pdf]

Supplementary Materials:

Table S1: Information of the access NO. of the *AGPAT* gene family used in this study

Gene	Species	Access NO.	Gene	Species	Access NO.
AGPAT1	buffalo	XP_025123832.1	AGPAT1	cattle	NP_803484.1
AGPAT2	buffalo	XP_025118206.3	AGPAT2	cattle	NP_001073733.1
AGPAT3	buffalo	XP_044803467.1	AGPAT3	cattle	NP_001033135.1
AGPAT4	buffalo	XP_044779956.1	AGPAT4	cattle	NP_001015537.1
AGPAT5	buffalo	XP_006050741.1	AGPAT5	cattle	NP_001069400.1
AGPAT6	buffalo	NP_001277775.1	AGPAT6	cattle	NP_001077138.1
AGPAT1	goat	NP_001272690.1	AGPAT1	camel	XP_010980526.2
AGPAT2	goat	XP_017911619.1	AGPAT2	camel	XP_031306729.1
AGPAT3	goat	XP_017907308.1	AGPAT3	camel	XP_031316519.1
AGPAT4	goat	XP_005685016.2	AGPAT4	camel	XP_010975048.1
AGPAT5	goat	XP_017897541.1	AGPAT5	camel	XP_031296229.1
AGPAT6	goat	XP_017897472.1	AGPAT6	camel	XP_032324696.1
AGPAT1	sheep	XP_042092820.1	AGPAT1	horse	XP_001492118.2
AGPAT2	sheep	XP_014949598.2	AGPAT2	horse	XP_023485077.1
AGPAT3	sheep	XP_014948165.1	AGPAT3	horse	XP_023486028.1
AGPAT4	sheep	XP_027828438.2	AGPAT4	horse	XP_001500501.1
AGPAT5	sheep	XP_027818539.1	AGPAT5	horse	XP_023486446.1
AGPAT6	sheep	XP_042097328.1	AGPAT6	horse	XP_023486358.1
AGPAT1	pig	NP_001028180.1	AGPAT1	human	NP_001358366.1
AGPAT2	pig	NP_001124006.1	AGPAT2	human	NP_006403.2
AGPAT3	pig	NP_001137172.1	AGPAT3	human	NP_001032642.1
AGPAT4	pig	NP_001137169.1	AGPAT4	human	NP_064518.1
AGPAT5	pig	NP_001137173.1	AGPAT5	human	NP_060831.2
AGPAT6	pig	NP_001138491.1	AGPAT6	human	NP_001350126.1
AGPAT1	mouse	NP_001156851.1	AGPAT4	mouse	NP_080920.2
AGPAT2	mouse	NP_080488.1	AGPAT5	mouse	NP_081068.1
AGPAT3	mouse	NP_001391799.1	AGPAT6	mouse	NP_061213.2

Table S2: The sequences of siRNA fragments used in this study

Genes	siRNA	sense（5'-3'）	antisense（5'-3'）
AGPAT1	si1	GCAACGUCGAGAACAUGAATT	UUCAUGUUCUCGACGUUGCTT
	si2	CCAUUGUGCCCAUCGUCAUTT	AUGACGAUGGGCACAAUGGTT
	si3	GCACUCCAUGCUCACUGUUTT	AACAGUGAGCAUGGAGUGCTT
AGPAT3	si1	GGAAGGAGCAUGCAGUCAUTT	AUGACUGCAUGCUCCUUCCTT
	si2	CCGGGACACUGUCAUCAAATT	UUUGAUGACAGUGUCCCGGTT
	si3	GCAGCUCAGUGGCUUCAUATT	UAUGAAGCCACUGAGCUGCTT
AGPAT4	si1	GCUCCAAGGUCCUGGCUAATT	UUAGCCAGGACCUUGGAGCTT
	si2	GCGGAUUCCUCUAGAAGAATT	UUCUUCUAGAGGAAUCCGCTT
	si3	GGACAGCAAGCAGAAACAUTT	AUGUUUCUGCUUGCUGUCCTT
NC	Si-NC	UUCUCCGAACGUGUCACGUTT	ACGUGACACGUUCGGAGAATT

Table S3: Primers used in this study for qRT-PCR

Genes	Primer Sequences（5′-3′）	Access NO.	Tm（℃）
AGPAT1	F: GGCGGGAGTCATCTTCATTG<br>R: GCATGGAGCCGTTGTGGTTT	XM_025268047.2	60
AGPAT2	F: GGGAGAAGCTCAAAGTGTGG<br>R: AGTAAACCACGGGGATGATG	XM_025262421.3	60
AGPAT3	F: GGCAGCAGTTCAAACCCACC<br>R: AAGCTGCCCCGACAAACCCC	XM_044947532.2	60
AGPAT4	F: GGTGAACAAGCAGCTCTTCC<br>R: TCCGTGTAGATGACGCACTC	XM_044924021.2	60
AGPAT5	F: CCCGCTGTATGGCTGTTATT<br>R: TTGAGCAGCAAATGCTTGAC	XM_006050679.3	60
AGPAT6	F: TGCCTTTCGACAGCCTGATT<br>R: GAAAATGGCGGGGACGATGA	NM_001290846.1	60
β-actin	F: CTGGCATTGTCATGGACTCTG<br>R: GCTCGGCTGTGGTGGTAAA	XM_025274489.3	60
RPS9	F: CCTCGACCAAGAGCTGAAG<br>R: CCTCCAGACCTCACGTTTGTTC	XM_006053433.4	60
SCD	F: GTTTCTGTTACACTTGGGAGC<br>R: GACCATTCAAAAACGTCATTC	NM_001290915.1	60
ACACA	F: CCTGGGTCTCCTATCTTTGTC<br>R: TAATCTTCTGATGCCTGCGTT	XM_025281124.3	60
FASN	F: TACTTCGTGGTCTTCTCCTCG<br>R: TTGTCTTTAGTGCCCTTCAGC	XM_006061793.4	60
GPAM	F: GATCAGACTGACTGGATG<br>R: GGATGTTGAGATTATTGC	XM_006043943.4	60
PPARY	F: GCTCCAAGAGTACCAAAGTG<br>R: GTCCTCCTGAAGAAACCCTT	XM_044933433.2	60
Lpin1	F: GTCTTTCTAGCTCTTGCC<br>R: CTCTTTCATCTTGTGTGG	XM_006073740.4	60
DGAT2	F: GTCCTGTCTTTCCTCGTGCT<br>R: CCTCCTGCCACCTTTCTT	XM_006045187.3	60
ACSL1	F: GCCATGCAGTCAGTGGAGGTG<br>R: CAAGGGTGTCGTAGAGGGGAA	XM_006075115.1	60
